# Mitochondrial Genome Comparison and Phylogenetic Variety of Four Morphologically Similar Bamboo Pests

**DOI:** 10.1002/ece3.70588

**Published:** 2024-11-20

**Authors:** Yue Ying, Wenhao Wang, Yan Li, Zhihong Li, Xinkang Zhao, Shouke Zhang, Jinping Shu, Zhenming Shen, Wei Zhang

**Affiliations:** ^1^ Research Institute of Subtropical Forestry Chinese Academy of Forestry Hangzhou China; ^2^ State Key Laboratory of Subtropical Silviculture, Zhejiang A & F University Hangzhou China; ^3^ Lin'an District Agriculture and Rural Bureau Hangzhou China

**Keywords:** bamboo snout moth, divergence time, mitochondrial genome, phylogeny, Pyraloidea

## Abstract

Bamboo snout moths (Lepidoptera, Crambidae) comprise the four species: *Eumorphobotys obscuralis*, *Circobotys aurealis*, *Demobotys pervulgalis*, and *Crypsiptya coclesalis*. These economically important insect pests of bamboo are widely distributed in tropical and subtropical regions. The lack of precise mitochondrial genetic data has impeded the development of effective identification techniques, accurate classification strategies, and targeted prevention and treatment strategies. In this study, we obtained the complete mitochondrial genome sequences of four bamboo snout moth species using high‐throughput sequencing. The mitogenomes were 15,103–15,349 bp in length and contained 13 protein‐coding genes, 22 transfer RNA genes (tRNAs), two ribosomal RNA genes (rRNAs), and a noncoding region (A + T rich element), consistent with previously studied Crambidae mitogenomes. We reconstructed the phylogenetic relationships among the four species using Bayesian inference and maximum likelihood methods. The moths that fed on bamboo were well clustered in a single clade. *Crypsiptya coclesalis* was most closely related to *D. pervulgalis*, while *E. obscuralis* was most closely related to *C. aurealis*. The divergence among the main lineages of 97 Lepidoptera species was reconstructed using an uncorrelated relaxed molecular clock. Analyses of the phylogenetic relationships and divergence times showed that the evolution of lepidopteran species has been closely related to that of their hosts. The data support the development of molecular identification techniques for the four species of bamboo snout moth, and our results provide a basis for targeted control strategies.

## Graphical abstract

In this study, we sequenced the mitochondrial genomes of four bamboo snout moth species collected in Hangzhou (Zhejiang, China) and evaluated their composition bias, relative synonymous codon usage (RSCU), and nucleotide composition along with their genome structure, phylogenetic relationships, and divergence times. Our study makes a significant contribution to the literature because our findings provide a theoretical basis for a better study of the biological characteristics of bamboo snout moths and the prevention and control of bamboo snout moths.

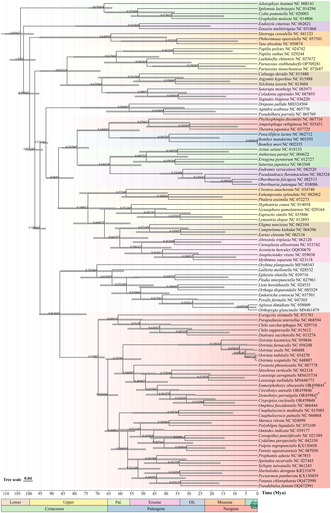

## 1 Introduction

Bamboo, a member of the Poaceae family, is a versatile plant with numerous socioeconomic applications. It is commonly used in landscaping, construction, papermaking, food production, fuel, and craft production (Dou, Yu, and Iwamatsu 2011; Shu and Wang 2015). Additionally, bamboo is involved in ecological stability and human food security (Basumatary et al. 2015; Sharma et al. 2021). Leaf‐rolling caterpillar larvae of the bamboo snout moths that damage bamboo plants, particularly the leaves of young bamboo (Zhang, Zheng, and Huang 2005). This is only observational data; however, as no relevant research studies have been conducted. Severe defoliation can lead to a decline in bamboo tree vitality, and even the death of bamboo groves when bamboo snout moth populations consume the leaves and shoots. Bamboo snout moths can significantly impact the growth of bamboo shoots and whips in the following year (Wu et al. 2018; Shen 2023). The most prevalent moth species in southern China are *Eumorphobotys obscuralis*, *Circobotys aurealis*, *Demobotys pervulgalis*, and *Crypsiptya coclesalis* (Zhang, Zheng, and Huang 2005). These species share similar characteristics and damage patterns, and their larval periods overlap. The populations of the bamboo snout moths have increased due to the intensification and expansion of bamboo forests (Cheng et al. 2012).

The mitochondrial genome—or, as it is sometimes called, the mitogenome—is an important tool for studying species evolution and phylogeny at various taxonomic levels (Li et al. 2015; Yingqi et al. 2019). Owing to its small size, maternal inheritance, high mutation rate, and lack of recombination (Chai, Du, and Zhai 2012; Cameron 2013), the mitochondrial genome is generally considered one of the most reliable and effective genetic markers in molecular phylogenetic studies. The mitogenome has been extensively researched in both plants and animals (Burger, Gray, and Lang 2003; Cameron 2013; Gualberto et al. 2014). The mitochondrial genome of insects is characteristically circular, ranging from 14 to 20 kb in size. The genome comprises one control region and 37 genes, including 13 protein‐coding genes, two rRNA genes, and 22 tRNA genes, with a highly variable number of tRNA genes (Chai, Du, and Zhai 2012; Sun et al. 2016). Analysis of the mitochondrial genome can provide a reliable basis for differentiating species that are difficult to classify using morphology (such as those in the Lepidoptera and Coleoptera) (Chai, Du, and Zhai 2012; Yingqi et al. 2019; Yang et al. 2020). The genes *coxI* and *coxII*, as well as gene orders, have been widely used in the identification of insect species and the study of population genetics (Hebert, Ratnasingham, and deWaard 2003; Rodrigues, Morelli, and Jansen 2017).

Mitochondrial genomes, which are extensively used in phylogenetic and population genetics, play a key role in identifying and classifying multiple insects and other organisms. Moreover, mitochondrial gene arrangement provides important information that aids in inferring the evolutionary relationships of insects (Cameron 2013). Phylogenetic analyses based on complete mitochondrial genomes provide better resolution of inferred phylogenetic trees compared with those based on partial gene fragments (Ruan et al. 2020; Xu, Yu, and Zhang 2020). Although bamboo snout moths are important lepidopteran pests, this group still contains many unreported species, and the published reports concerning bamboo snout moths do not include mitochondrial genome analysis.

Bamboo snout moths affect the development of the bamboo economy. Consequently, this study examined the mitochondrial genomes of four bamboo snout moth species collected in Hangzhou (Zhejiang, China) by evaluating their composition bias, relative synonymous codon usage (RSCU), and nucleotide composition. The phylogenetic relationships of bamboo moths within Lepidoptera were reconstructed based on 13 protein‐coding genes (PCGs) in the mitochondrial genome. The tRNA gene families (as well as their secondary structures) were compared with those of other sequenced lepidopteran species. The divergence times of the four bamboo moths were estimated, and selective pressure analyses provided genetic information and a reference for an indepth study of the biological characteristics of bamboo snout moths and possible strategies for pest management.

## 2 Materials and Methods

### 2.1 Sample Collection and DNA Extraction

Four species of bamboo snout moths (*E. obscuralis*, *C. aurealis*, *D. pervulgalis*, and *C. coclesalis*) were obtained live from a bamboo grove in Hangzhou, Zhejiang, China (Sampling permit certificate ID of Zhejiang Provincial Forest Pest Control Station: ZJS‐SFZZ‐ZM‐2021‐1025.). The specimens were preserved in 100% ethanol and stored at −80°C prior to DNA isolation. Genomic DNA was isolated from one individual of each species, using a genomic DNA extraction kit (Aidlab Co., Beijing, China). The quality of the isolated DNA was tested using 1% agarose gel electrophoresis. The DNA was then used to amplify the complete mitochondrial genome.

### 2.2 DNA Sequencing and Genome Assembly

Illumina PE library construction and high‐throughput sequencing of the mitogenomes of the four moth species were performed using an Illumina HiSeq X Ten platform at Personalbio Technology Co. Ltd. (Shanghai, China). The sequencing libraries, which had an average insert size of ~ 400 bp, generated ~ 5 Gb of raw data. The removal of low‐quality, contaminated reads, along with high ‘N’ ratio sequences and adapters, resulted in high‐quality data. The clean reads from each of the four species were separately assembled *de novo* using NOVOPlasty software (https://github.com/ndierckx/NOVOPlasty) (Dierckxsens, Mardulyn, and Smits 2017).

### 2.3 Gene Annotation and Sequence Analysis

Annotation of the mitochondrial genomes of the four newly assembled bamboo snout moth species was performed using the MITOS web server (http://mitos2.bioinf.uni‐leipzig.de/index.py) based on the Pyraloidea code (Liu et al. 2021). The start and stop codons were verified using previously published Pyraloidea mitochondrial genomes as references (Chai, Du, and Zhai 2012; Dai et al. 2018; Yang et al. 2020; Liu et al. 2021). The circular genomes of the four species were visualized using Brig v.0.95 (Alikhan et al. 2011). MEGA 11.0 (Tamura, Stecher, and Kumar 2021), TBtools (Chen et al. 2020a), and RStudio were used to analyze the nucleotide composition, PCGs, tRNA genes, and rRNA genes of each species' mitogenome. A and T content values as well as RSCU and codon usage of PCGs were also determined. Base skew values were calculated using the formulas AT skew = (A − T)/(A + T) and GC skew = (G − C)/(G + C). The evolutionary adaptation was verified by estimating the rates of nonsynonymous (Ka) and synonymous (Ks) substitutions in the mitogenomes of 35 species of Crambidae using DnaSP 6.1203 (Rozas et al. 2017).

### 2.4 Phylogenetic Analysis

Evolutionary relationships were reconstructed using the protein‐coding genes (PCGs) from 99 mitogenomes, including the four newly sequenced species (*E. obscuralis*, *C. aurealis*, *D. pervulgalis*, and *C. coclesalis*) and two outgroups ( *Anopheles gambiae* and *Drosophila melanogaster* ) (see Table S1). Multiple alignments of the concatenated nucleotide sequences of the 13 PCGs were performed using MAFFT v.7.520 (Katoh and Standley 2013). These sequences were used for phylogenetic analyses, and phylogenetic trees were constructed using two analytical approaches, namely maximum likelihood (ML) and Bayesian inference (BI). Ultrafast likelihood bootstrapping with 1000 bootstrap replicates was applied to reconstruct a consensus tree. The best substitution model was determined using the AIC in ModelFinder for MrBayes. The analysis of business intelligence involved two Markov Chain Monte Carlo (MCMC) runs. Each run consisted of 2,000,000 generations, with a sampling frequency of every 1000 generations. The first 25% of each run was discarded as a burn‐in.

### 2.5 Divergence Time Estimation

The divergence times of the four species were estimated at the nucleotide level (13 PCGs) using MCMC Tree in PAML to perform Bayesian estimation. Soft fossil constraints were employed under various molecular clock models (Puttick 2019). The divergence times of *Adoxophyes honmai*/*Spilonota lechriaspis*, *Luehdorfia chinensis*/*Telchinia issoria*, *Drepana pallida*/ *Bombyx mori*, and *Chilo sacchariphagus*/*Ostrinia furnacalis* were estimated to be 63.9–74.3 million years ago (Mya), 69.3–118.8 Mya, 91.6–93.7 Mya, and 66.2–71.4 Mya, respectively. The split divergence times of, Endromis versicolora/Oberthueria jiatongae (24.2–45.1 Mya) and *Bombyx mori* /*Bombyx mandarina* (0.0041 Mya) were estimated using a prior. The Markov chain was run twice for 100 million generations, with sampling every 1000 generations and a burn‐in of the initial 25% of the samples. Chain convergence was confirmed using Tracer v.1.6, and many of the values exceeded an effective sample size (ESSs) of 200. The phylogenetic tree and divergence times were visualized using FigTree v.1.4.3 software. Graphics were created using Chiplot (Xie et al. 2023).

## 3 Results

### 3.1 Gene Organization

**FIGURE 1:**
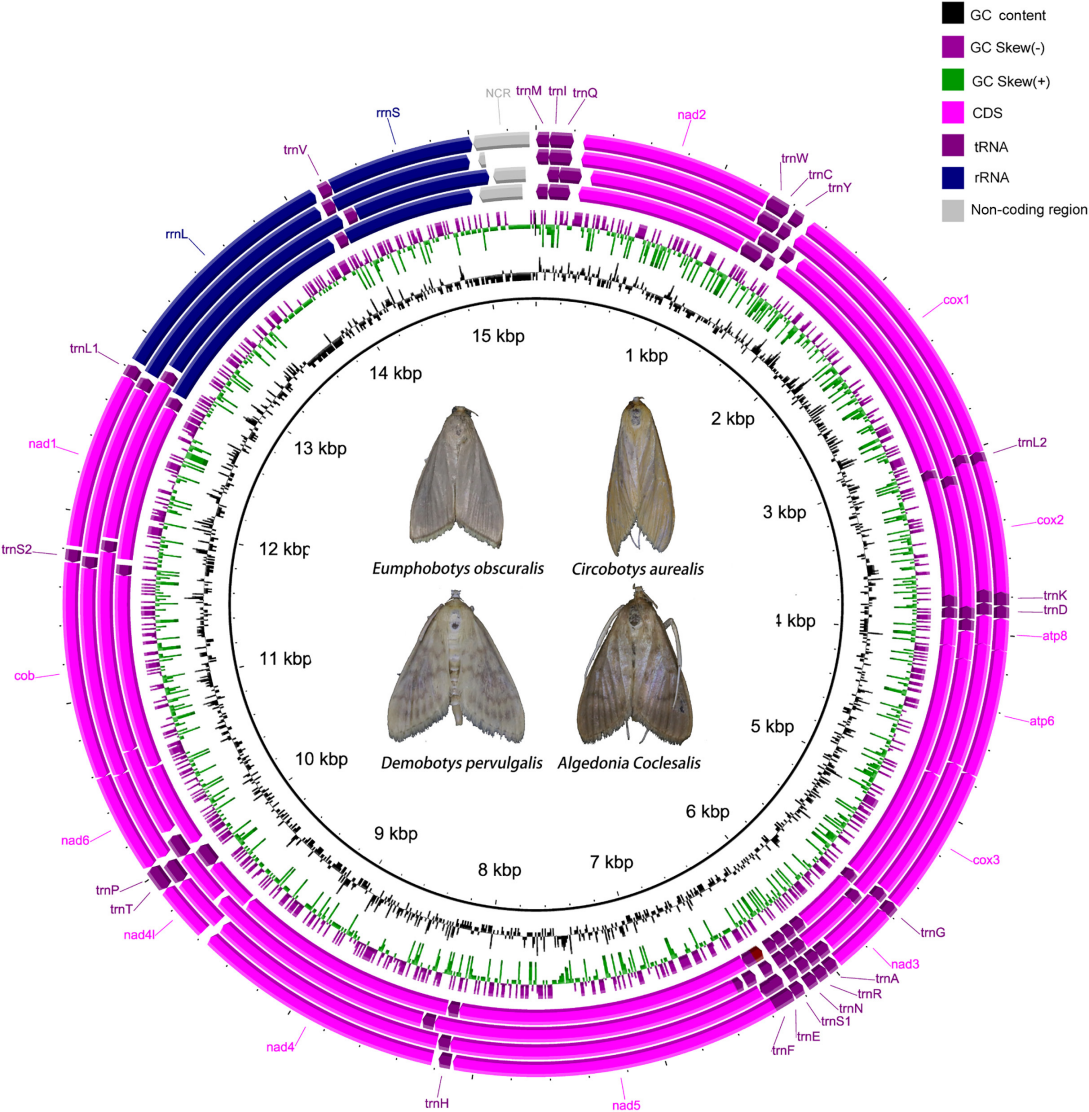
Gene map of the complete mitogenomes of *C. coclesalis*, *C. aurealis*, *D. pervulgalis*, and *E. obscuralis* (from the inner to outer). The ring indicates the gene arrangement and distribution.

In this study, we present the complete mitochondrial genomes of the four bamboo snout moths with lengths of 15,349 bp for *E. obscuralis*, 15,288 bp for *C. aurealis*, 15,103 bp for *D. pervulgalis*, and 15,301 bp for *C. coclesalis* (GenBank accession: OR459845–OR459848). The mitochondrial genome composition and structure of these four species were highly concordant. Each mitochondrial genome contained 13 PCGs (*cox1‐3*, *cytb*, *nad1‐6*, *nad4l*, *atp6*, and *atp8*), 22 tRNA genes, two RNA genes (*rrnL* and *rrnS*), and one major noncoding region (Figure 1; Table S2). The difference in the length of the mitogenomes was mainly associated with the variation in length of the noncoding regions. As in the mitochondrial genomes of other Lepidoptera (Dai et al. 2018; Liu et al. 2021), the four mitochondrial genomes all had a relatively compact structure, where the longest intergenic sequence (51 bp) was found between *nad4* and *nad4l* of *E. obscuralis*; the longest overlap sequence, 35 bp, was found between *cox2* and *trnK* (Table S2). The genome structures of the four species were identical to those of other Pyraloidea taxa (Dai et al. 2018; Liu et al. 2021), without gene rearrangements; this finding may be related to their life histories and biological characteristics. The overall A + T contents of *E. obscuralis*, *C. aurealis*, *D. pervulgalis*, and *C. coclesalis* were 81.05%, 79.60%, 79.96%, and 78.73%, respectively (Table S3). The A and T contents were high, signifying a codon usage bias toward A and T. An apparent bias against G and C was shown by the low G and C contents of the four species. Furthermore, all four mitochondrial genomes showed positive AT skew and negative GC skew, indicating the occurrence of more A nucleotides than T nucleotides and fewer G nucleotides than C nucleotides.

### 3.2 Protein‐Coding Genes

The four newly sequenced mitogenomes contained 13 PCGs, with *atp8* being the smallest (162–171 bp) and *nad5* being the largest (1738–1750 bp), which was consistent with other Crambidae mitogenomes (Liu et al. 2021). Nine of the 13 PCGs were encoded by the minority strand (N‐strand). Six PCGs (*cox2*, *atp8*, *nad5*, *nad6*, *cob*, and *nad1*) differed in size, and three PCGs (*nad2*, *cox2*, and *nad6*) had inconsistent start conditions among the four moths. The initiation codons for the *nad2* gene of *C. coclesalis* and the nad6 gene of *E. obscuralis* were ATC and ATA, respectively, while the other two species used ATT. All other protein‐coding genes (PCGs) used consistent initiation codons (Table S2). While 13 PCGs were terminated with TAA codons, *nad4l* in *E. obscuralis* and *D. pervulgalis* were terminated with TAG. All protein‐coding genes (PCGs) on the N‐strand showed negative GC skews, whereas the four PCGs on the J‐strand displayed positive GC skews. This result was consistent with previous studies (Chai, Du, and Zhai 2012; Yang et al. 2018). The maximum negative and positive GC skews were observed in *atp8* and *nad4l*, respectively. The mitochondrial genomes of *E. obscuralis*, *C. aurealis*, *D. pervulgalis*, and *C. coclesalis* encoded 3746, 3737, 3739, and 3735 amino acids, respectively. The four species had the highest frequency of Leu, Ile, Phe, and Ser amino acids (Figure 1). Cys was the least common amino acid, with all frequencies below 0.80%. This pattern was consistent with previous reports on two Pyraloidea species, *Diaphania indica* and *Omiodes indicata* (Dai et al. 2018; Yang et al. 2018).

**FIGURE 2:**
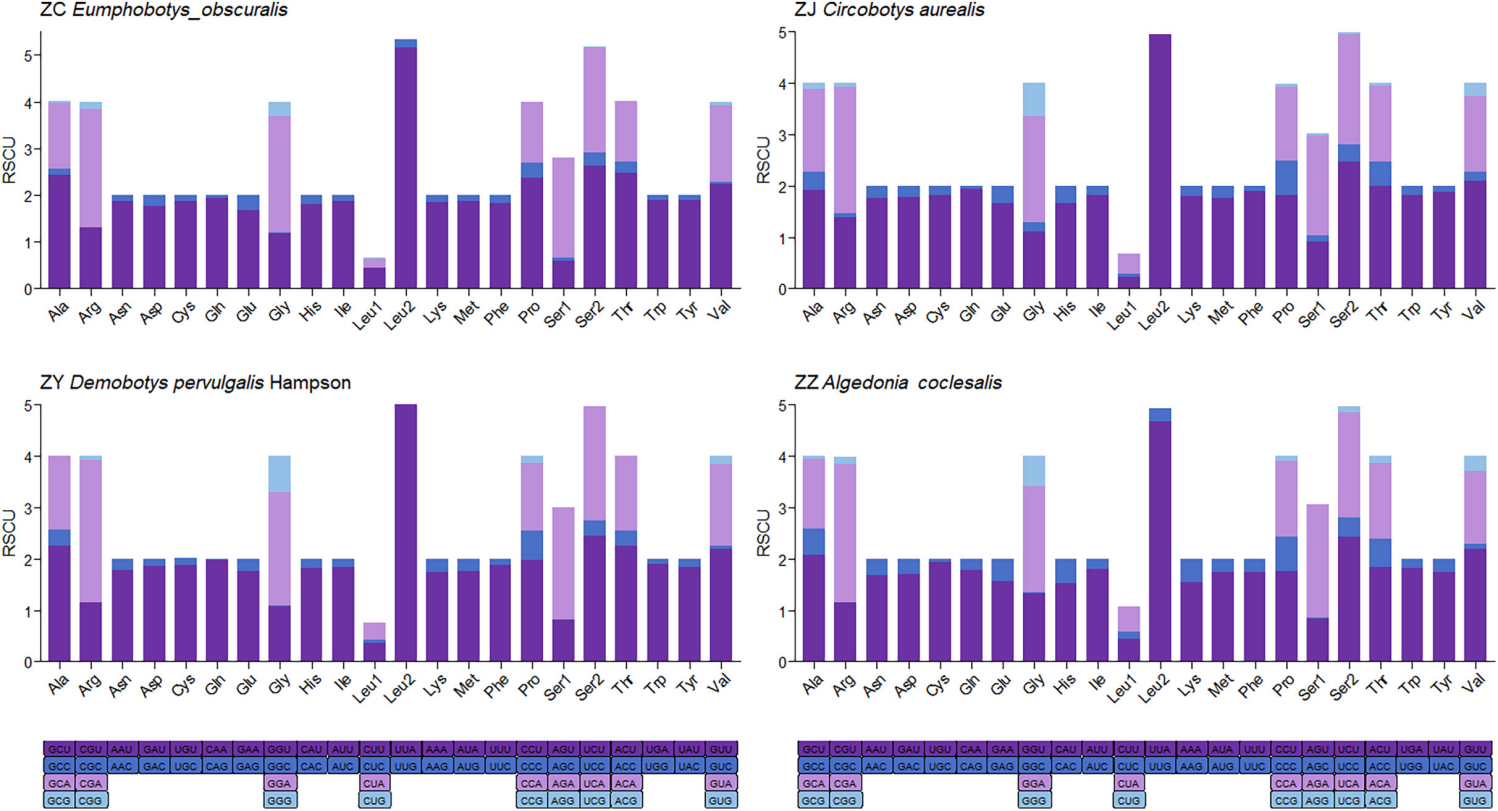
Relative synonymous codon usage (RSCU) in the mitochondrial genomes of four species.

**FIGURE 3:**
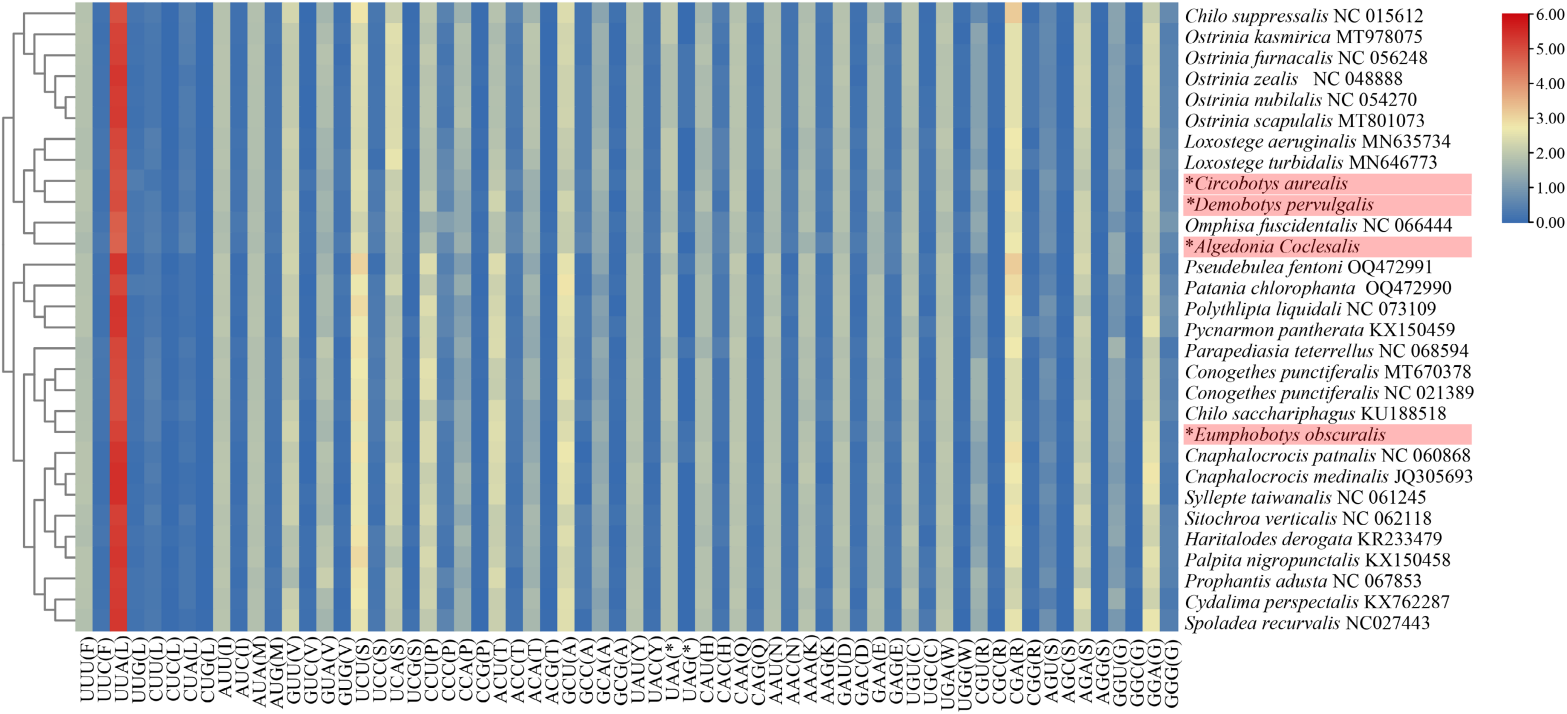
Heatmap of codon usage for protein‐coding genes in Pyraloidea. Red and blue colors in the heatmap indicate high and low absolute correlation, respectively. Species marked with “*” and highlighted in red are bamboo snout moths of this study. The species in the rows of the heat map are sorted by the corresponding cluster tree based on codon usage. The columns indicate 64 codons of invertebrate mitochondrion. Termination codon: A, alanine; C, cysteine; D, aspartic acid; E, glutamic acid; F, phenylalanine; G, glycine; H, histidine; I, isoleucine; K, lysine; L, leucine; M, methionine; N, asparagine; P, proline; Q, glutamine; R, arginine; S, serine; T, threonine; V, valine; W, tryptophan; Y, tyrosine.

The RSCU in the mitochondrial genomes of the four moths indicated similar but slightly different patterns (Figure 2). The most frequent codon was UUA (L) in all four species, followed by UCU(S) in *E. obscuralis* and *C. aurealis* and CGA(R) in *D. pervulgalis* and *C. coclesalis*. A dendrogram based on codon usage showed *C. aurealis* and *D. pervulgalis* clustered together, indicating a close relationship between these two species (Figure 3). *C. coclesalis* was closer to *Omphisa fuscidentalis* (NC066444), and *E. obscuralis* was closer to *Chilo sacchariphagus* (KU188518).

### 3.3 Transfer RNA Genes, Ribosomal RNA Genes, and Noncoding Regions

**FIGURE 4:**
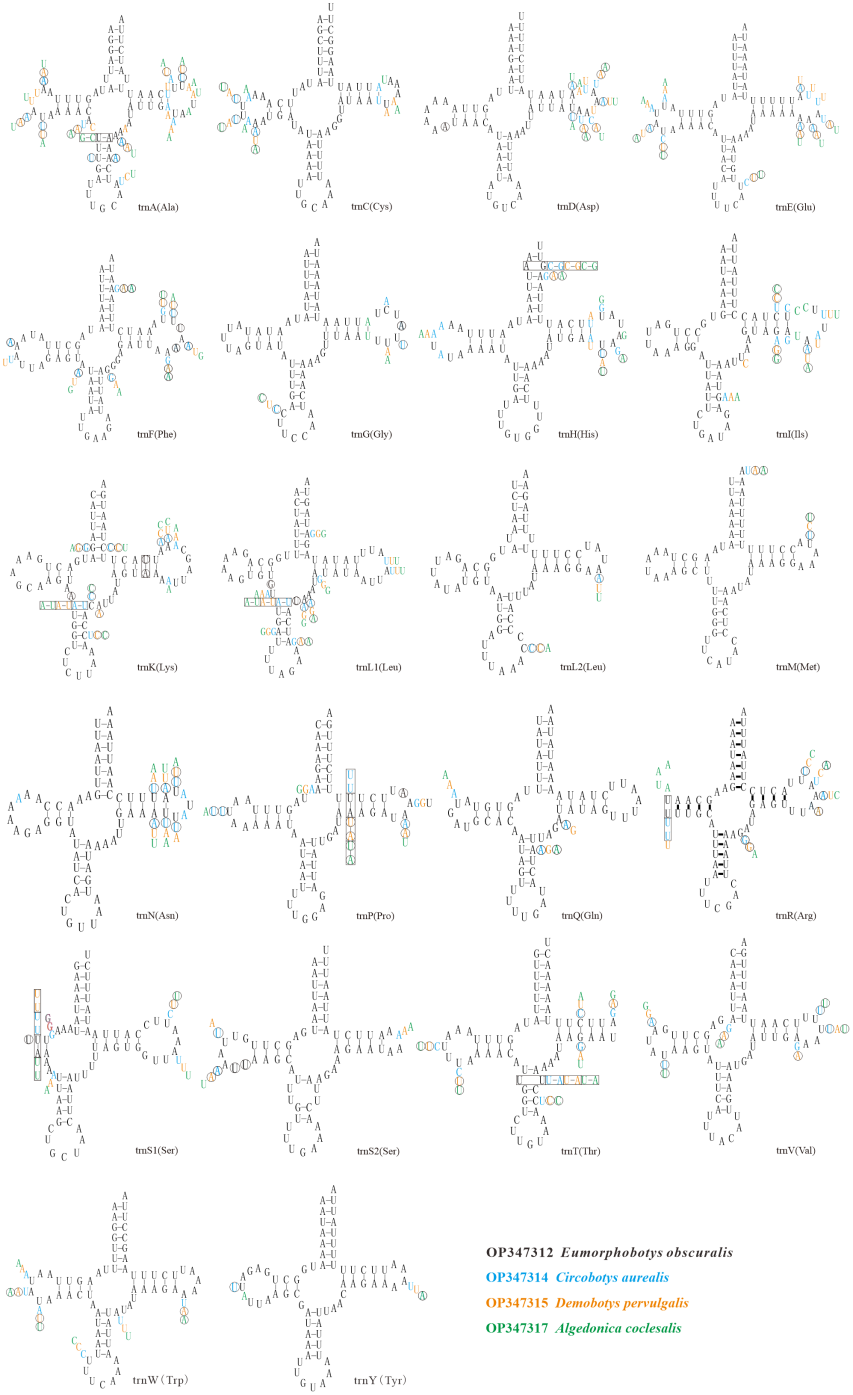
Predicted secondary cloverleaf structures for the 22 transfer RNA genes of four bamboo moths.

There were no significant differences in the positions of the 22 tRNA genes among the moths. The lengths of the tRNA genes ranged from 64 bp (*trnR* of *C. aurealis* and *D. pervulgalis*) to 72 bp (*trnA* of *D. pervulgalis*) (Table S1). Fourteen tRNAs were encoded by the N‐strand and eight by the J‐strand. The tRNAs had standard anticodons and could be folded into the typical cloverleaf structure, except for trnS1, which lacked a dihydrouridine (DHU) arm in all the four sequenced mitogenomes, and *trnR*, which lacked a DHU arm in *E. obscuralis*, *D. pervulgalis*, *C. Coclesalis*. The lack of a DHU arm has been observed in trnS1 of several other Pyraloidea species, including *Cnaphalocrocis medinalis* (Chai, Du, and Zhai 2012); however, the lack of a DHU arm in *trnR* is uncommon (Chai, Du, and Zhai 2012; Dai et al. 2018; Yang et al. 2018; Yang, Chen, and Dong 2022). The nucleotide substitution model of the tRNAs is presented in Figure 4. Base differences among the four species were observed in all 22 tRNA genes. *TrnM* and *trnL2* were highly conserved (identity = 100%), and the identities of *trnQ*, *trnY*, and *trnS2* were over 95%. Nucleotide substitution was higher in *trnA*. The TΨC arm and variable loop regions were more variable in nucleotide sequences than in other regions (Zhang et al. 2018).

Among the nucleotide substitution models of the tRNAs, G‐U was the most common noncanonical base pair, followed by U‐U and C‐U. This was consistent with the result of several previous studies on the mitogenomes of invertebrates (Dai et al. 2018). The lengths of the rrnL and rrnS genes were 1282–1292 bp and 773–781 bp, respectively. The A and T contents were greater than the G and C contents in the RNA genes. Both rRNA genes were separated by trnV and located in the usual position (Chai, Du, and Zhai 2012; Dai et al. 2018; Yang et al. 2018; Liu et al. 2021). The control regions of all four species were located between the trnM and rrnS, like the pattern found in *Diaphania indica* and *Omiodes indicate* (Dai et al. 2018; Yang et al. 2018). However, the control region of the four species was smaller (a sequencing error occurred in the region that may have resulted in failed PCR amplification), and Sanger sequencing of the complete mitogenome for the putative secondary structure of the AT‐rich region indicated several strong stem‐loop structures (Dai et al. 2018; Yang et al. 2018; Liu et al. 2021).

### 3.4 Selective Pressure Analysis

**FIGURE 5:**
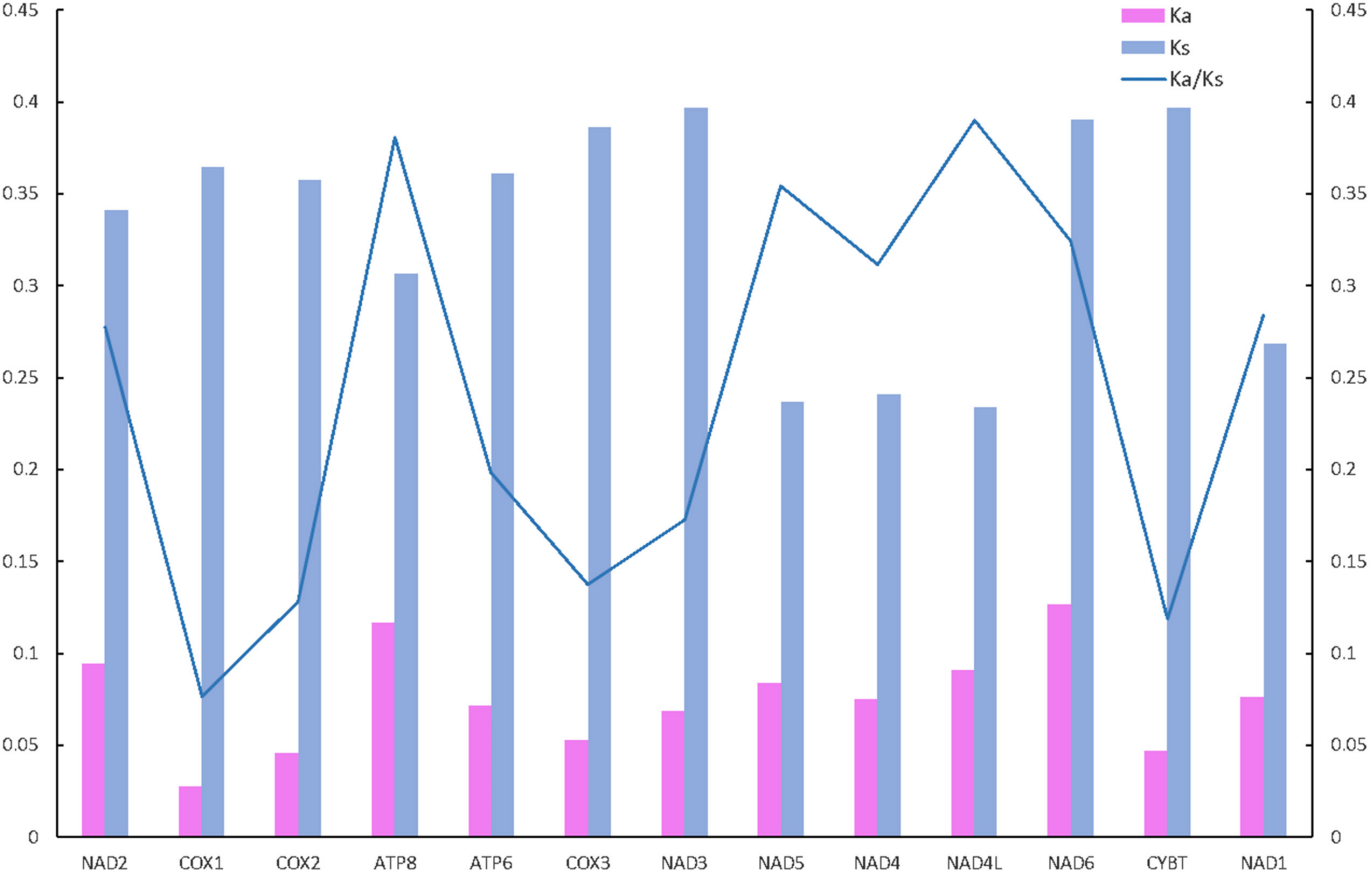
Blue line indicates the mean pairwise divergence of the Ka/Ks ratio for 13 PCGs among 35 Crambidae mitochondrial genomes. The 35 species are listed in Table S1. The pink and blue boxes indicate the number of nonsynonymous substitutions per nonsynonymous site (Ka) and the number of synonymous substitutions per synonymous site (Ks), respectively.

Generally, nucleotide diversity is used to identify regions with high nucleotide divergence. The analysis can provide guidelines for the selection of species‐ or group‐specific markers for molecular evolutionary studies (Ding et al. 2023). Mitogenomes from 31 Pyraloidea species were used to examine the evolutionary relationships among the moths and to identify selective pressure based on the nonsynonymous to synonymous substitution (Ka/Ks) ratio. The Ka/Ks ratio was < 1 for all PCGs, indicating that the mutations yielded synonymous substitutions (Hurst 2002). These results suggest that all 13 PCGs in the 31 Pyraloidea mitogenomes evolved under purifying selection and thus were suitable for investigating phylogenetic relationships within Pyraloidea (Figure 5). The results also showed different evolutionary rates for the 13 PCGs. The *CoxI* gene had the lowest Ka/Ks ratio among the studied genes and showed little change in amino acids, which is consistent with previous research findings and supports the extensive use of *CoxI* as a molecular marker for species identification and phylogenetic analysis (Astrin et al. 2016).

### 3.5 Phylogenetic Analysis

**FIGURE 6:**
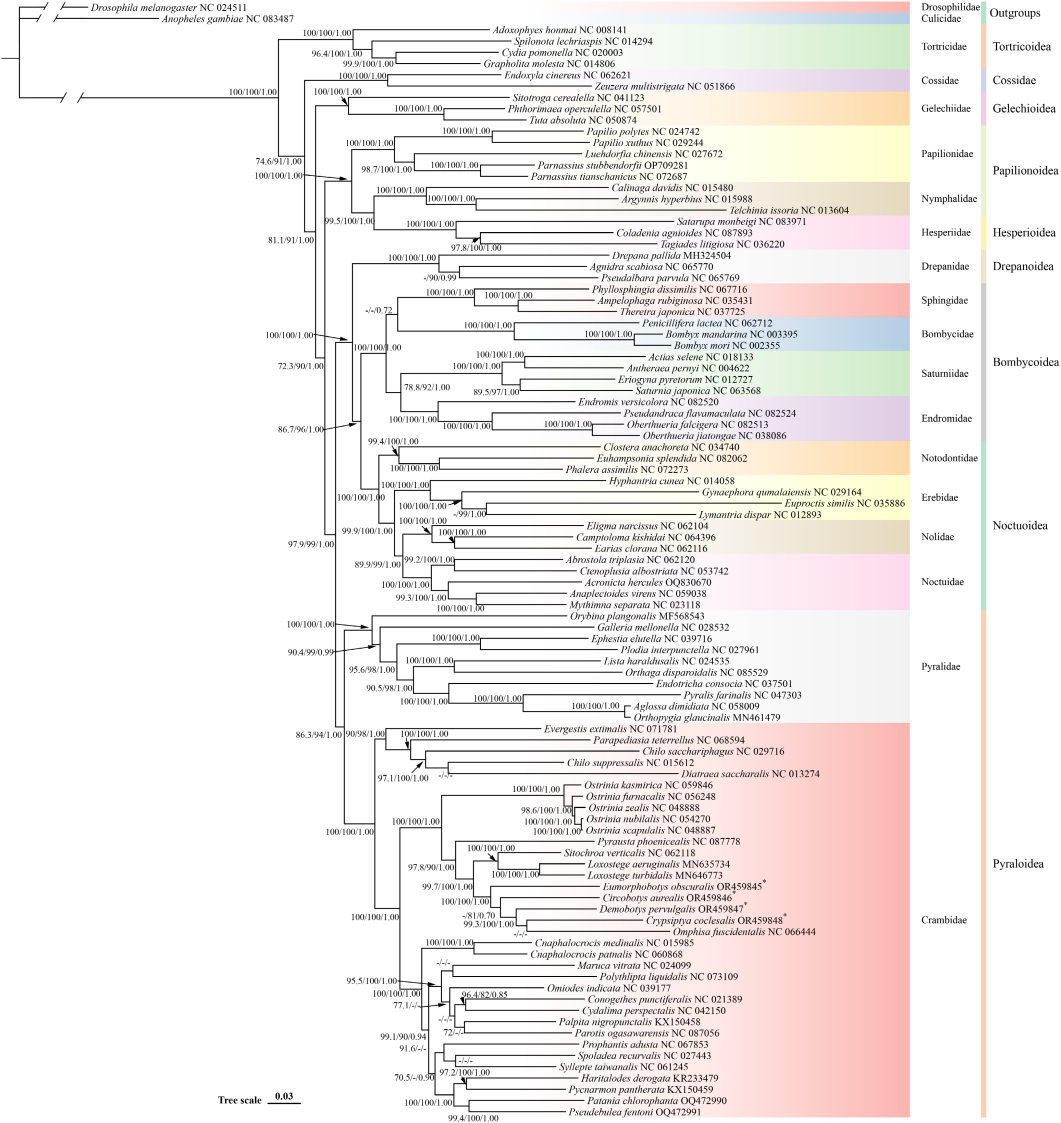
Phylogenetic tree inferred using Bayesian inference (BI) and maximum likelihood (ML) methods based on concatenated sequences of 13 PCGs from 99 mitogenomes. The sequences of two species ( *Anopheles gambiae* NC 083487 and *Drosophila melanogaster* NC 024511) were chosen as the outgroups. Species marked with “*” and highlighted in red are bamboo snout moths of this study. The number at each node is the bootstrap probability (SH‐aLRT support (%)/ultrafast bootstrap support (%)/posterior probability).

Phylogenetic analyses were performed based on the concatenated alignment of 13 PCGs covering 97 species from 17 families of Lepidoptera, with corresponding sequences from *A. gambiae* (NC 083487) and *D. melanogaster* (NC 024511) as outgroups (Figure 6). Maximum likelihood (ML) and Bayesian inference (BI) analyses produced almost identical topologies, with strong bootstrap and posterior probability values. Nevertheless, the Bayesian tree exhibited potential long‐branch attraction (Feng et al. 2021) in the branches of the four bamboo moths. To increase the reliability of the results, we combined the ML and BI methods to obtain a consistent evolutionary tree.

Phylogenetic analysis indicated that the four bamboo snout moths were clustered on the same branch. The posterior probability value was > 0.7, while bootstrap values were > 85%. The branching orders of the four bamboo snout moths slightly differed due to the increasing abundance of Crambideae species. The clade in which *C. coclesalis*, *D. pervulgalis*, *C. aurealis*, and *E. obscuralis* were clustered contained five species, all of which rely on bamboo as their host and food source. Furthermore, other species of Crambideae Pyraloidea showed host aggregation, that is, species with the same host clustered in the same clade. Therefore, the host may be an important factor in differentiating the Crambideae.

### 3.6 Divergence Times

**FIGURE 7:**
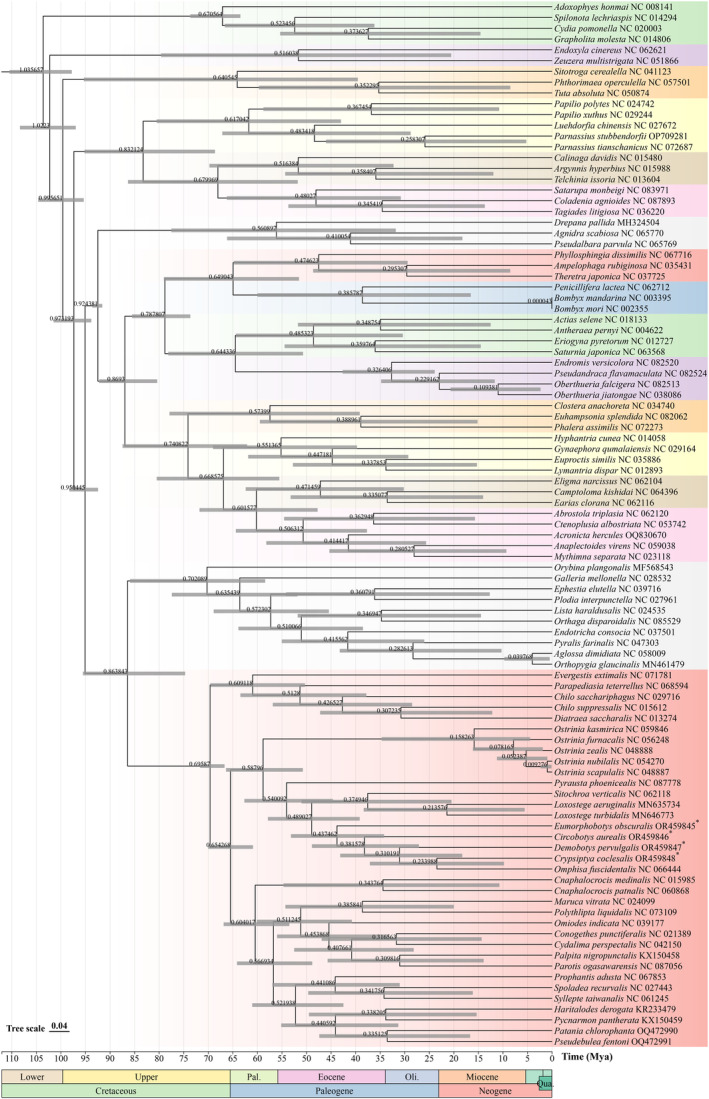
Divergence time estimation inferred via Bayesian relaxed dating methods (BEAST) based on the nucleotide sequences of 13 PCGs. Species marked with “*” are bamboo snout moths of this study.

The time‐calibrated phylogeny indicated that the divergence times of *Adoxophyes honmai*/*Spilonota lechriaspis* (Wheat and Wahlberg 2013; Fagua et al. 2018), *Luehdorfia chinensis*/*Telchinia issoria* (Kawahara et al. 2019; Vasilikopoulos et al. 2020), *Drepana pallida*/ *Bombyx mori* (Kawahara et al. 2019; Barber et al. 2022), and *Chilo sacchariphagus*/*Ostrinia furnacalis* were 67.06, 83.21, 92.44, and 69.59 Mya (Figure 7), respectively, in agreement with previous studies (Kumar et al. 2022). The divergence time of *Bombyx mori* /*Bombyx mandarina* was 0.0042 Mya, close to the result (0.0041 Mya) of Sun et al. (2012). The divergence time of *Endromis versicolora*/*Oberthueria jiatongae* was 32.64 Mya, within the range of 24.2–45.1 Mya (Kawahara and Barber 2015). Thus, our divergence time results are reliable to a certain extent. The divergence time of the four bamboo snout moths from the other Crambidae was estimated as 48.90 Mya (95% highest posterior density [HPD] interval = 39.12–57.82 Mya), within the Oligocene (Chattian, ∼ 25 Mya) and Eocene (Ypresian, ∼ 52 Mya) of the Paleogene period. During this period, the climate began to change from extremely warm to cold, resulting in seasonal changes in plant composition (Wang et al. 2010).

Deciduous tree species, which can better adapt to drastic temperature changes, began to have an advantage over evergreen species (Wilf 2000; Chen et al. 2020b). Within the Poaceae, the deciduous Bamboideae originated in the Middle Eocene (fossil‐estimated differentiation time across the entire Eocene and up to the Late Cretaceous), which coincides with the divergence times of the five bamboo snout moths and other Crambidae (Prasad et al. 2011). Therefore, changes in the environment and host could be key reasons for the initial differentiation of bamboo snout moths. There are some errors in the data set that may have resulted from different methods of classification and confirmation of the fossil record and different levels of experience and expertise. Among the five bamboo snout moth species, *E. obscuralis* and *C. aurealis* diverged at 26.78 Mya, and *C. coclesalis*, *D. pervulgalis*, and *Omphisa fuscidentalis* at 28.95 Mya. There was only a gap of ~ 9.05 million years between *C. coclesalis* and the other two species (*D. pervulgalis* and *O. fuscidentalis*). The divergence times of the five species were very similar, a result that may explain their comparable life histories and physical characteristics.

## 4 Conclusions

Using high‐throughput sequencing, we obtained mitogenome sequences from four bamboo snout moths, namely *E. obscuralis*, *C. aurealis*, *D. pervulgalis*, and *C. coclesalis*, with lengths of 15,349 bp, 15,288 bp, 15,103 bp, and 15,301 bp, respectively. Each mitogenome was comprised of a single control region, two rRNAs, 13 PCGs, and 22 tRNAs. The genome size, gene order, and nucleotide composition of these four mitogenomes were similar to those previously reported species of family Crambidae. Most PCGs were initiated with an ATG codon and terminated with a TAA codon. The Ka/Ks ratio indicated that PCGs in these Crambidae species were subjected to purifying selection. Phylogenetic trees not only contributed to the scientific classification but also demonstrated the importance of host species for the differentiation of the Pyraloidea family. This study provides information on the genetic characteristics, phylogenetic relationships, and evolution of Crambidae, as well as a basis for pest control. The four bamboo snout moth species differentiated in the Middle Paleogene and Early Neogene, and their evolution may be related to the climate change events that altered their living environments and host plants.

## Author Contributions

**Yue Ying:** data curation (equal), software (equal). **Wenhao Wang:** formal analysis (equal), validation (equal). **Yan Li:** formal analysis (equal), validation (equal). **Zhihong Li:** data curation (equal), software (equal). **Xinkang Zhao:** data curation (equal), software (equal). **Shouke Zhang:** conceptualization (equal), writing – review and editing (equal). **Jinping Shu:** funding acquisition (equal), writing – review and editing (equal). **Zhenming Shen:** conceptualization (equal), funding acquisition (equal), writing – review and editing (equal). **Wei Zhang:** project administration (equal), writing – review and editing (equal).

## Conflicts of Interest

The authors declare no conflicts of interest.

## Supporting information


Table S1.

## Data Availability

Sampling permit certificate ID of Zhejiang Provincial Forest Pest Control Station: ZJS‐SFZZ‐ZM‐2021‐1025. The data that support the findings of this study are openly available in NCBI: OR459845–OR459848.
