# Supplementary material for: Mitochondrial Genome Comparison and Phylogenetic Variety of Four Morphologically Similar Bamboo Pests
Source: Ecol Evol. 2024 Nov 20;14(11):e70588. doi: 10.1002/ece3.70588 (PMC11578632; doi:10.1002/ece3.70588)
Supplement: Supplementary file 1 — Table S1. [file ECE3-14-e70588-s001.docx]

**Table S1. information for the polygenomic analysis.**

| Organism | Superfamily | Family | Size (bp) | Accession number |
| --- | --- | --- | --- | --- |
| *Penicillifera lactea* | Bombycoidea | Bombycidae | 15402 | NC_062712.1 |
| *Bombyx mandarina* | Bombycoidea | Bombycidae | 15928 | NC_003395.1 |
| *Bombyx mori* | Bombycoidea | Bombycidae | 15643 | NC_002355.1 |
| *Endoxyla cinereus* | Cossoidea | Cossidae | 15285 | NC_062621.1 |
| *Zeuzera multistrigata* | Cossoidea | Cossidae | 15320 | NC_051866.1 |
| *Crypsiptya coclesalis** | Pyraloidea | Crambidae | 15301 | OR459848.1 **in the study** |
| *Demobotys pervulgalis** | Pyraloidea | Crambidae | 15103 | OR459847.1 **in the study** |
| *Circobotys aurealis** | Pyraloidea | Crambidae | 15288 | OR459846.1 **in the study** |
| *Eumorphobotys obscuralis** | Pyraloidea | Crambidae | 15348 | OR459845.1 **in the study** |
| *Pseudebulea fentoni* | Pyraloidea | Crambidae | 15266 | OQ472991.1 |
| *Patania chlorophanta* | Pyraloidea | Crambidae | 15298 | OQ472990.1 |
| *Pyrausta phoenicealis* | Pyraloidea | Crambidae | 15281 | NC_087778.1 |
| *Parotis ogasawarensis* | Pyraloidea | Crambidae | 15261 | NC_087056.1 |
| *Polythlipta liquidalis* | Pyraloidea | Crambidae | 15305 | NC_073109.1 |
| *Evergestis extimalis* | Pyraloidea | Crambidae | 15301 | NC_071781.1 |
| *Parapediasia teterrellus* | Pyraloidea | Crambidae | 15368 | NC_068594.1 |
| *Prophantis adusta* | Pyraloidea | Crambidae | 15689 | NC_067853.1 |
| *Omphisa fuscidentalis* | Pyraloidea | Crambidae | 15347 | NC_066444.1 |
| *Sitochroa verticalis* | Pyraloidea | Crambidae | 15275 | NC_062118.1 |
| *Syllepte taiwanalis* | Pyraloidea | Crambidae | 15264 | NC_061245.1 |
| *Cnaphalocrocis patnalis* | Pyraloidea | Crambidae | 15305 | NC_060868.1 |
| *Ostrinia kasmirica* | Pyraloidea | Crambidae | 15214 | NC_059846.1 |
| *Ostrinia furnacalis* | Pyraloidea | Crambidae | 15241 | NC_056248.1 |
| *Ostrinia nubilalis* | Pyraloidea | Crambidae | 14838 | NC_054270.1 |
| *Ostrinia zealis* | Pyraloidea | Crambidae | 15208 | NC_048888.1 |
| *Ostrinia scapulalis* | Pyraloidea | Crambidae | 15311 | NC_048887.1 |
| *Cydalima perspectalis* | Pyraloidea | Crambidae | 15232 | NC_042150.1 |
| *Omiodes indicata* | Pyraloidea | Crambidae | 15367 | NC_039177.1 |
| *Chilo sacchariphagus* | Pyraloidea | Crambidae | 15378 | NC_029716.1 |
| *Spoladea recurvalis* | Pyraloidea | Crambidae | 15273 | NC_027443.1 |
| *Maruca vitrata* | Pyraloidea | Crambidae | 15385 | NC_024099.1 |
| *Conogethes punctiferalis* | Pyraloidea | Crambidae | 15355 | NC_021389.1 |
| *Cnaphalocrocis medinalis* | Pyraloidea | Crambidae | 15388 | NC_015985.1 |
| *Chilo suppressalis* | Pyraloidea | Crambidae | 15395 | NC_015612.1 |
| *Diatraea saccharalis* | Pyraloidea | Crambidae | 15490 | NC_013274.1 |
| *Loxostege turbidalis* | Pyraloidea | Crambidae | 15240 | MN646773.1 |
| *Loxostege aeruginalis* | Pyraloidea | Crambidae | 15339 | MN635734.1 |
| *Pycnarmon pantherata* | Pyraloidea | Crambidae | 15545 | KX150459.1 |
| *Palpita nigropunctalis* | Pyraloidea | Crambidae | 15226 | KX150458.1 |
| *Haritalodes derogata* | Pyraloidea | Crambidae | 15253 | KR233479.1 |
| *Agnidra scabiosa* | Drepanoidea | Drepanidae | 15461 | NC_065770.1 |
| *Pseudalbara parvula* | Drepanoidea | Drepanidae | 15304 | NC_065769.1 |
| *Drepana pallida* | Drepanoidea | Drepanidae | 15339 | MH324504.1 |
| *Pseudandraca flavamaculata* | Bombycoidea | Endromidae | 15569 | NC_082524.1 |
| *Endromis versicolora* | Bombycoidea | Endromidae | 15574 | NC_082520.1 |
| *Oberthueria falcigera* | Bombycoidea | Endromidae | 15657 | NC_082513.1 |
| *Oberthueria jiatongae* | Bombycoidea | Endromidae | 15673 | NC_038086.1 |
| *Euproctis similis* | Noctuoidea | Erebidae | 15437 | NC_035886.1 |
| *Gynaephora qumalaiensis* | Noctuoidea | Erebidae | 15753 | NC_029164.1 |
| *Hyphantria cunea* | Noctuoidea | Erebidae | 15481 | NC_014058.1 |
| *Lymantria dispar* | Noctuoidea | Erebidae | 15569 | NC_012893.1 |
| *Phthorimaea operculella* | Gelechioidea | Gelechiidae | 15263 | NC_057501.1 |
| *Tuta absoluta* | Gelechioidea | Gelechiidae | 15290 | NC_050874.1 |
| *Sitotroga cerealella* | Gelechioidea | Gelechiidae | 15312 | NC_041123.1 |
| *Coladenia agnioides* | Hesperioidea | Hesperiidae | 15308 | NC_087893.1 |
| *Satarupa monbeigi* | Hesperioidea | Hesperiidae | 15338 | NC_083971.1 |
| *Tagiades litigiosa* | Hesperioidea | Hesperiidae | 15359 | NC_036220.1 |
| *Acronicta hercules* | Noctuoidea | Noctuidae | 15321 | OQ830670.1 |
| *Abrostola triplasia* | Noctuoidea | Noctuidae | 15363 | NC_062120.1 |
| *Anaplectoides virens* | Noctuoidea | Noctuidae | 15358 | NC_059038.1 |
| *Ctenoplusia albostriata* | Noctuoidea | Noctuidae | 15284 | NC_053742.1 |
| *Mythimna separata* | Noctuoidea | Noctuidae | 15332 | NC_023118.1 |
| *Camptoloma kishidai* | Noctuoidea | Nolidae | 15353 | NC_064396.1 |
| *Earias clorana* | Noctuoidea | Nolidae | 15561 | NC_062116.1 |
| *Eligma narcissus* | Noctuoidea | Nolidae | 15346 | NC_062104.1 |
| *Euhampsonia splendida* | Noctuoidea | Notodontidae | 15397 | NC_082062.1 |
| *Phalera assimilis* | Noctuoidea | Notodontidae | 15757 | NC_072273.1 |
| *Clostera anachoreta* | Noctuoidea | Notodontidae | 15456 | NC_034740.1 |
| *Argynnis hyperbius* | Papilionoidea | Nymphalidae | 15156 | NC_015988.1 |
| *Calinaga davidis* | Papilionoidea | Nymphalidae | 15267 | NC_015480.1 |
| *Telchinia issoria* | Papilionoidea | Nymphalidae | 15245 | NC_013604.1 |
| *Parnassius stubbendorfii* | Papilionoidea | Papilionidae | 15348 | OP709281.1 |
| *Parnassius tianschanicus* | Papilionoidea | Papilionidae | 15432 | NC_072687.1 |
| *Papilio xuthus* | Papilionoidea | Papilionidae | 15359 | NC_029244.1 |
| *Luehdorfia chinensis* | Papilionoidea | Papilionidae | 16028 | NC_027672.1 |
| *Papilio polytes* | Papilionoidea | Papilionidae | 15256 | NC_024742.1 |
| *Orthaga disparoidalis* | Pyraloidea | Pyralidae | 15272 | NC_085529.1 |
| *Aglossa dimidiata* | Pyraloidea | Pyralidae | 15225 | NC_058009.1 |
| *Pyralis farinalis* | Pyraloidea | Pyralidae | 15204 | NC_047303.1 |
| *Ephestia elutella* | Pyraloidea | Pyralidae | 15346 | NC_039716.1 |
| *Endotricha consocia* | Pyraloidea | Pyralidae | 15201 | NC_037501.1 |
| *Galleria mellonella* | Pyraloidea | Pyralidae | 15320 | NC_028532.1 |
| *Plodia interpunctella* | Pyraloidea | Pyralidae | 15264 | NC_027961.1 |
| *Lista haraldusalis* | Pyraloidea | Pyralidae | 15213 | NC_024535.1 |
| *Orthopygia glaucinalis* | Pyraloidea | Pyralidae | 15229 | MN461479.1 |
| *Orybina plangonalis* | Pyraloidea | Pyralidae | 14823 | MF568543.1 |
| *Saturnia japonica* | Bombycoidea | Saturniidae | 15369 | NC_063568.1 |
| *Actias selene* | Bombycoidea | Saturniidae | 15236 | NC_018133.1 |
| *Eriogyna pyretorum* | Bombycoidea | Saturniidae | 15327 | NC_012727.1 |
| *Antheraea pernyi* | Bombycoidea | Saturniidae | 15566 | NC_004622.2 |
| *Phyllosphingia dissimilis* | Bombycoidea | Sphingidae | 15259 | NC_067716.1 |
| *Theretra japonica* | Bombycoidea | Sphingidae | 15399 | NC_037725.1 |
| *Ampelophaga rubiginosa* | Bombycoidea | Sphingidae | 15282 | NC_035431.1 |
| *Cydia pomonella* | Tortricoidea | Tortricidae | 15253 | NC_020003.2 |
| *Grapholita molesta* | Tortricoidea | Tortricidae | 15717 | NC_014806.1 |
| *Spilonota lechriaspis* | Tortricoidea | Tortricidae | 15368 | NC_014294.1 |
| *Adoxophyes honmai* | Tortricoidea | Tortricidae | 15680 | NC_008141.1 |
| *Anopheles gambiae* | Culicoidea | Culicidae | 15363 | NC_083487.1 |
| *Drosophila melanogaster* | Ephydroidea | Drosophilidae | 19524 | NC_024511.2 |

**Note: All datas from NCBI, except bamboo snout moths “in the study”.**

**Table S2. Annotion of the mitogenomes of *E. obscuralis*, *C. aurealis*, *D. pervulgalis*, *A. coclesalis***

| Gene | Position(start-end) | | | | Size(bp) | | | | Initiation codon | | | | Stop codon | | | | Strand | Intergenic nucleotide | | | |
| --- | --- | --- | --- | --- | --- | --- | --- | --- | --- | --- | --- | --- | --- | --- | --- | --- | --- | --- | --- | --- | --- |
|  | ZC | ZJ | ZY | ZZ | ZC | ZJ | ZY | ZZ | ZC | ZJ | ZY | ZZ | ZC | ZJ | ZY | ZZ |  | ZC | ZJ | ZY | ZZ |
| *trnM* | 1-68 | 63-130 | 1-68 | 1-68 | 68 | 68 | 68 | 68 |  |  |  |  |  |  |  |  | N |  | -3 | -1 | 1 |
| *TrnI* | 69-133 | 128-194 | 68-132 | 70-137 | 65 | 67 | 65 | 68 |  |  |  |  |  |  |  |  | N | -3 | -3 | -3 | -3 |
| *TrnQ* | 131-199 | 192-260 | 130-198 | 135-203 | 69 | 69 | 69 | 69 |  |  |  |  |  |  |  |  | J | 49 | 49 | 49 | 48 |
| *nad2* | 249-1,262 | 310-1,323 | 248-1,261 | 252-1,265 | 1,014 | 1,014 | 1,014 | 1,014 | ATT | ATT | ATT | ATC | TAA | TAA | TAA | TAA | N | 7 | 6 | 7 | 7 |
| *trnW* | 1,270-1,337 | 1,330-1,397 | 1,269-1,335 | 1,273-1,340 | 68 | 68 | 67 | 68 |  |  |  |  |  |  |  |  | N | -8 | -8 | -8 | -8 |
| *trnC* | 1,330-1,394 | 1,390-1,457 | 1,328-1,393 | 1,333-1,401 | 65 | 68 | 66 | 69 |  |  |  |  |  |  |  |  | J | 25 | 38 | 10 | 21 |
| *trnY* | 1,420-1,486 | 1,496-1,562 | 1,404-1,469 | 1,423-1,490 | 67 | 67 | 66 | 68 |  |  |  |  |  |  |  |  | J | 37 | 36 | 38 | 41 |
| *cox1* | 1,524-3,026 | 1,599-3,101 | 1,508-3,010 | 1,532-3,034 | 1,503 | 1,503 | 1,503 | 1,503 | ATT | ATT | ATT | ATT | TAA | TAA | TAA | TAA | N | -5 | -5 | -5 | -5 |
| *trnL2* | 3,022-3,088 | 3,097-3,163 | 3,006-3,072 | 3,030-3,096 | 67 | 67 | 67 | 67 |  |  |  |  |  |  |  |  | N | -15 | -15 | -18 | -18 |
| *cox2* | 3,074-3,805 | 3,149-3,880 | 3,055-3,789 | 3,079-3,813 | 732 | 732 | 735 | 735 | ATC | ATC | ATT | ATT | TAA | TAA | TAA | TAA | N | -35 | -35 | -35 | -35 |
| *trnK* | 3,771-3,841 | 3,846-3,916 | 3,755-3,825 | 3,779-3,849 | 71 | 71 | 71 | 71 |  |  |  |  |  |  |  |  | N |  |  |  | -1 |
| *trnD* | 3,842-3,908 | 3,917-3,983 | 3,826-3,892 | 3,849-3,915 | 67 | 67 | 67 | 67 |  |  |  |  |  |  |  |  | N |  |  |  |  |
| *atp8* | 3,909-4,079 | 3,984-4,148 | 3,893-4,054 | 3,916-4,077 | 171 | 165 | 162 | 162 | ATC | ATC | ATC | ATC | TAA | TAA | TAA | TAA | N | -7 | -7 | -7 | -7 |
| *atp6* | 4,073-4,747 | 4,142-4,816 | 4,048-4,722 | 4,071-4,745 | 675 | 675 | 675 | 675 | ATG | ATG | ATG | ATG | TAA | TAA | TAA | TAA | N | -1 | -1 | -1 | -1 |
| *cox3* | 4,747-5,535 | 4,816-5,604 | 4,722-5,510 | 4,745-5,533 | 789 | 789 | 789 | 789 | ATG | ATG | ATG | ATG | TAA | TAA | TAA | TAA | N | 4 | 11 | 6 | 6 |
| *trnG* | 5,540-5,606 | 5,616-5,681 | 5,517-5,582 | 5,540-5,605 | 67 | 66 | 66 | 66 |  |  |  |  |  |  |  |  | N | -3 | -3 | -3 | -3 |
| *nad3* | 5,604-5,960 | 5,679-6,035 | 5,580-5,936 | 5,603-5,959 | 357 | 357 | 357 | 357 | ATA | ATA | ATA | ATA | TAA | TAA | TAA | TAA | N | 8 | 2 | 10 | 2 |
| *trnA* | 5,969-6,033 | 6,038-6,104 | 5,947-6,018 | 5,962-6,032 | 65 | 67 | 72 | 71 |  |  |  |  |  |  |  |  | N | 2 | -1 | -1 | 2 |
| *trnR* | 6,036-6,100 | 6,104-6,167 | 6,018-6,081 | 6,035-6,100 | 65 | 64 | 64 | 66 |  |  |  |  |  |  |  |  | N | 1 | 1 | 4 | 4 |
| *trnN* | 6,102-6,167 | 6,169-6,235 | 6,086-6,151 | 6,105-6,170 | 66 | 67 | 66 | 66 |  |  |  |  |  |  |  |  | N | 17 | 29 | 10 |  |
| *trnS1* | 6,185-6,249 | 6,265-6,330 | 6,162-6,227 | 6,171-6,236 | 65 | 66 | 66 | 66 |  |  |  |  |  |  |  |  | N | 1 | 41 | 28 | 28 |
| *trnE* | 6,251-6,317 | 6,372-6,440 | 6,256-6,324 | 6,265-6,330 | 67 | 69 | 69 | 66 |  |  |  |  |  |  |  |  | N | -2 | 13 |  | -2 |
| *trnF* | 6,316-6,386 | 6,454-6,522 | 6,325-6,392 | 6,329-6,396 | 71 | 69 | 68 | 68 |  |  |  |  |  |  |  |  | J | -17 | -17 | 3 | -3 |
| *nad5* | 6,370-8,112 | 6,506-8,254 | 6,396-8,145 | 6,394-8,131 | 1,743 | 1,749 | 1,750 | 1,738 | ATT | ATT | ATT | ATT | TAA | TAA | T(AA) | T(AA) | J | 10 |  |  |  |
| *trnH* | 8,123-8,188 | 8,255-8,324 | 8,146-8,211 | 8,132-8,198 | 66 | 70 | 66 | 67 |  |  |  |  |  |  |  |  | J | 33 | 5 | 6 | 5 |
| *nad4* | 8,222-9,562 | 8,330-9,670 | 8,218-9,558 | 8,204-9,544 | 1,341 | 1,341 | 1,341 | 1,341 | ATG | ATG | ATG | ATG | TAA | TAA | TAA | TAA | J | 51 | 25 | 33 | 12 |
| *nad4l* | 9,614-9,907 | 9,696-9,989 | 9,592-9,885 | 9,557-9,850 | 294 | 294 | 294 | 294 | ATG | ATG | ATG | ATG | TAG | TAA | TAG | TAA | J | 2 | 2 | 2 | 2 |
| *trnT* | 9,910-9,975 | 9,992-10,057 | 9,888-9,953 | 9,853-9,918 | 66 | 66 | 66 | 66 |  |  |  |  |  |  |  |  | N |  |  |  |  |
| *trnP* | 9,976-10,042 | 10,058-10,123 | 9,954-10,019 | 9,919-9,984 | 67 | 66 | 66 | 66 |  |  |  |  |  |  |  |  | J | 5 | 32 | 32 | 32 |
| *nad6* | 10,048-10,581 | 10,156-10,662 | 10,052-10,558 | 10,017-10,523 | 534 | 507 | 507 | 507 | ATA | ATT | ATT | ATT | TAA | TAA | TAA | TAA | N | -1 | -1 | -1 | 5 |
| *Cob* | 10,581-11,729 | 10,662-11,807 | 10,558-11,706 | 10,529-11,680 | 1,149 | 1,146 | 1,149 | 1,152 | ATG | ATG | ATG | ATG | TAA | TAA | TAA | TAA | N | 2 | -2 | -2 | -2 |
| *trnS2* | 11,732-11,798 | 11,806-11,871 | 11,705-11,770 | 11,679-11,743 | 67 | 66 | 66 | 65 |  |  |  |  |  |  |  |  | N | 21 | 19 | 21 | 35 |
| *nad1* | 11,820-12,755 | 11,891-12,829 | 11,792-12,730 | 11,779-12,714 | 936 | 939 | 939 | 936 | ATG | ATG | ATG | ATG | TAA | TAA | TAA | TAA | J | 1 | 1 | 1 | 1 |
| *trnL1* | 12,757-12,824 | 12,831-12,899 | 12,732-12,800 | 12,716-12,784 | 68 | 69 | 69 | 69 |  |  |  |  |  |  |  |  | J | 26 | 27 | 28 | 22 |
| *rrnL* | 12,851-14,140 | 12,927-14,208 | 12,829-14,120 | 12,807-14,097 | 1,290 | 1,282 | 1,292 | 1,291 |  |  |  |  |  |  |  |  | J | 29 | 26 | 27 | 28 |
| *trnV* | 14,170-14,236 | 14,235-14,301 | 14,148-14,212 | 14,126-14,192 | 67 | 67 | 65 | 67 |  |  |  |  |  |  |  |  | J |  |  |  |  |
| *rrnS* | 14,237-15,012 | 14,302-15,082 | 14,213-14,985 | 14,193-14,971 | 776 | 781 | 773 | 779 |  |  |  |  |  |  |  |  | J | 5 | 22 | 47 | 40 |
| OH | 15,018-15,312 | 15,105-15,288 | 15,033-15,070 | 15,012-15,267 | 295 | 184 | 38 | 256 |  |  |  |  |  |  |  |  | N | 36 | -1 | 32 | 33 |

**Table S3. Nucleotide composition of the mitogenomes of four bamboo snout moth species**

| Region | A% | | C% | | G% | | T% | | AT_skew | | GC_skew | |
| --- | --- | --- | --- | --- | --- | --- | --- | --- | --- | --- | --- | --- |
|  | ZC | ZJ | ZC | ZJ | ZC | ZJ | ZC | ZJ | ZC | ZJ | ZC | ZJ |
| Whole genome | 40.99 | 40.67 | 11.47 | 12.74 | 7.49 | 7.66 | 40.06 | 38.93 | 0.011 | 0.022 | -0.21 | -0.249 |
| nad2 | 35.9 | 35.5 | 8.58 | 9.66 | 5.82 | 6.21 | 49.7 | 48.62 | -0.161 | -0.156 | -0.192 | -0.217 |
| cox1 | 32.27 | 32.34 | 14.24 | 15.44 | 14.04 | 13.84 | 39.45 | 38.39 | -0.1 | -0.086 | -0.007 | -0.055 |
| cox2 | 36.75 | 36.61 | 12.02 | 13.25 | 10.79 | 11.2 | 40.44 | 38.93 | -0.048 | -0.031 | -0.054 | -0.084 |
| atp8 | 43.27 | 42.42 | 7.6 | 7.27 | 2.34 | 3.03 | 46.78 | 47.27 | -0.039 | -0.054 | -0.529 | -0.412 |
| atp6 | 34.52 | 33.33 | 13.33 | 14.52 | 7.7 | 9.04 | 44.44 | 43.11 | -0.126 | -0.128 | -0.268 | -0.233 |
| cox3 | 33.33 | 32.95 | 13.94 | 14.96 | 12.42 | 12.42 | 40.3 | 39.67 | -0.095 | -0.092 | -0.058 | -0.093 |
| nad3 | 34.45 | 34.73 | 12.04 | 12.04 | 6.44 | 6.44 | 47.06 | 46.78 | -0.155 | -0.148 | -0.303 | -0.303 |
| nad5 | 32.99 | 32.42 | 5.97 | 6.12 | 11.76 | 13.21 | 49.28 | 48.26 | -0.198 | -0.196 | 0.327 | 0.367 |
| nad4 | 32.66 | 31.54 | 6.11 | 6.34 | 12.08 | 14.17 | 49.14 | 47.95 | -0.201 | -0.206 | 0.328 | 0.382 |
| nad4l | 29.93 | 29.59 | 3.74 | 4.08 | 12.93 | 13.27 | 53.4 | 53.06 | -0.282 | -0.284 | 0.551 | 0.529 |
| nad6 | 38.39 | 39.05 | 8.8 | 9.27 | 4.87 | 4.93 | 47.94 | 46.75 | -0.111 | -0.09 | -0.288 | -0.306 |
| cob | 34.38 | 34.03 | 13.58 | 15.36 | 9.75 | 10.12 | 42.3 | 40.49 | -0.103 | -0.087 | -0.164 | -0.205 |
| nad1 | 29.81 | 28.43 | 7.37 | 7.14 | 14.1 | 15.12 | 48.72 | 49.31 | -0.241 | -0.268 | 0.313 | 0.359 |
| rrnL | 41.55 | 40.09 | 5.04 | 5.15 | 10.7 | 12.01 | 42.71 | 42.75 | -0.014 | -0.032 | 0.36 | 0.4 |
| rrnS | 43.43 | 43.79 | 4.9 | 4.74 | 9.66 | 9.48 | 42.01 | 42 | 0.017 | 0.021 | 0.327 | 0.333 |
| OH | 42.71 | 41.85 | 2.71 | 2.17 | 1.02 | 1.09 | 53.56 | 54.89 | -0.113 | -0.135 | -0.455 | -0.333 |
| Region | A% | | C% | | G% | | T% | | AT_skew | | GC_skew | |
|  | ZZ | ZY | ZZ | ZY | ZZ | ZY | ZZ | ZY | ZZ | ZY | ZZ | ZY |
| Whole genome | 40.44 | 40.41 | 13.41 | 12.29 | 7.87 | 7.75 | 38.28 | 39.55 | 0.027 | 0.011 | -0.26 | -0.226 |
| nad2 | 34.42 | 34.91 | 11.74 | 9.57 | 6.51 | 6.21 | 47.34 | 49.31 | -0.158 | -0.171 | -0.286 | -0.212 |
| cox1 | 31.4 | 31.87 | 16.7 | 14.64 | 14.17 | 14.17 | 37.72 | 39.32 | -0.091 | -0.105 | -0.082 | -0.016 |
| cox2 | 36.73 | 36.19 | 12.93 | 13.47 | 11.29 | 10.88 | 39.05 | 39.46 | -0.031 | -0.043 | -0.067 | -0.106 |
| atp8 | 43.83 | 43.83 | 8.64 | 9.26 | 3.09 | 1.85 | 44.44 | 45.06 | -0.007 | -0.014 | -0.474 | -0.667 |
| atp6 | 35.11 | 34.07 | 15.41 | 14.07 | 9.04 | 8.3 | 40.44 | 43.56 | -0.071 | -0.122 | -0.261 | -0.258 |
| cox3 | 33.21 | 32.57 | 16.6 | 13.69 | 12.55 | 12.8 | 37.64 | 40.94 | -0.063 | -0.114 | -0.139 | -0.033 |
| nad3 | 33.33 | 35.85 | 15.69 | 11.2 | 7 | 6.16 | 43.98 | 46.78 | -0.138 | -0.132 | -0.383 | -0.29 |
| nad5 | 31.76 | 33.31 | 6.27 | 6.11 | 13.58 | 12.4 | 48.39 | 48.17 | -0.207 | -0.182 | 0.368 | 0.34 |
| nad4 | 31.62 | 32.96 | 6.56 | 6.41 | 14.02 | 12.98 | 47.8 | 47.65 | -0.204 | -0.182 | 0.362 | 0.338 |
| nad4l | 27.55 | 29.59 | 4.42 | 3.74 | 15.99 | 13.27 | 52.04 | 53.4 | -0.308 | -0.287 | 0.567 | 0.56 |
| nad6 | 38.07 | 38.66 | 12.43 | 10.26 | 5.92 | 5.33 | 43.59 | 45.76 | -0.068 | -0.084 | -0.355 | -0.316 |
| cob | 33.85 | 34.12 | 16.15 | 14.36 | 10.24 | 10.1 | 39.76 | 41.43 | -0.08 | -0.097 | -0.224 | -0.174 |
| nad1 | 28.21 | 29.39 | 7.37 | 7.35 | 15.6 | 14.8 | 48.82 | 48.46 | -0.268 | -0.245 | 0.358 | 0.337 |
| rrnL | 40.59 | 41.1 | 5.34 | 5.19 | 11.7 | 10.99 | 42.37 | 42.72 | -0.021 | -0.019 | 0.373 | 0.359 |
| rrnS | 42.88 | 42.82 | 4.88 | 4.79 | 10.14 | 9.7 | 42.11 | 42.69 | 0.009 | 0.002 | 0.35 | 0.339 |
| OH | 47.66 | 50 | 2.73 | 0 | 0.39 | 0 | 49.22 | 50 | -0.016 | 0 | -0.75 | 0 |
